# Enhanced oral bioavailability of an etoposide multiple nanoemulsion incorporating a deoxycholic acid derivative–lipid complex

**DOI:** 10.1080/10717544.2020.1837293

**Published:** 2020-10-27

**Authors:** Saurav Kumar Jha, Hee-Soo Han, Laxman Subedi, Rudra Pangeni, Jee Young Chung, Seho Kweon, Jeong Uk Choi, Youngro Byun, Yong-Hee Kim, Jin Woo Park

**Affiliations:** a Department of Biomedicine, Health & Life Convergence Sciences, BK21 Four, Mokpo National University, Muan-gun, Republic of Korea; b Department of Bioengineering, Institute for Bioengineering and Biopharmaceutical Research, BK 21 Plus Future Biopharmaceutical Human Resources Training and Research Team, Hanyang University, Seoul, Republic of Korea; c College of Pharmacy and Natural Medicine Research Institute, Mokpo National University, Muan-gun, Republic of Korea; d Department of Bioengineering, Hanyang University, Seoul, Republic of Korea; e Department of Molecular Medicine and Biopharmaceutical Science, Graduate School of Convergence Science and Technology, College of Pharmacy, Seoul National University, Seoul, Republic of Korea; f College of Pharmacy, Chonnam National University, Gwangju, Republic of Korea

**Keywords:** Etoposide, nanoemulsion, permeability, oral bioavailability, bile acid transporter-mediated uptake, oral absorption

## Abstract

In this study, a system for oral delivery of etoposide (ETP) was designed to avoid the problems associated with low and variable bioavailability of a commercially available ETP emulsion comprised of polyethylene glycol, glycerol, and citric acid anhydrous. ETP was complexed with low-molecular-weight methylcellulose (ETP/LMC) and loaded into a water-in-oil-in-water multiple nanoemulsion to formulate an ETP/LMC-nanoemulsion (ELNE). To further enhance the oral bioavailability, an ionic complex formed by anionic lipid 1,2-didecanoyl-sn-glycero-3-phosphate (sodium salt) and cationic *N*^α^-deoxycholyl-l-lysyl-methylester was incorporated into ELNE, yielding ELNE#7. As expected, ELNE#7 showed 4.07- and 2.25-fold increases in artificial membrane and Caco-2/HT29-MTX-E12 permeability (*P_app_*), respectively, resulting in 224% greater oral bioavailability compared with the commercially available ETP emulsion. In contrast, inhibition of clathrin- and caveola-mediated endocytosis, macropinocytosis, and bile acid transporters by chlorpromazine, genistein, amiloride, and actinomycin D in Caco-2/HT-29-MTX-E12 monolayers reduced the *P_app_* by 45.0%, 20.5%, 28.8%, and 31.1%, respectively. These findings suggest that these routes play important roles in enhancing the oral absorption of ELNE#7. In addition, our mechanistic study suggested that P-glycoprotein did not have an inhibitory effect on the permeation of ELNE#7. Notably, ELNE#7 showed significantly enhanced toxicity in LLC and A549 cells compared with ETP-E. These observations support the improved oral absorption of ETP in ELNE#7, suggesting that it is a better alternative than ETP emulsion.

## 1. Introduction

The preference for oral anticancer drugs has increased over the last decade because of improved patient compliance, lower costs, proven efficacy, lack of infusion-related problems, and flexibility of the dosage design. However, oral administration of antineoplastic agents is often compromised by their limited and variable bioavailability due to poor aqueous solubility and permeability of the drugs. To address these issues, they are generally solubilized in an excess of organic solvent/co-solvent and are administered as the maximum tolerated dose via the intravenous route (Ghadi & Dand, 2017). Etoposide (ETP), a podophyllotoxin-derived topoisomerase II inhibitor, has significant therapeutic activity against a wide range of cancers, such as childhood leukemia, testicular tumors, small cell lung cancer, and metastatic breast cancer (Toffoli et al., 2004). Moreover, the efficacy of ETP is dose-schedule-dependent, and best responses are observed with prolonged oral treatment. However, systemic exposure to ETP following oral dosing is highly variable. Therefore, widespread use of oral ETP is limited because of the variation in therapeutic response, with some patients experiencing suboptimal tumor cytotoxicity, while others may be at risk of excess cytotoxicity (Joel et al., 1995; Hande et al., 1999).

The low oral bioavailability of ETP is due mainly to its poor aqueous solubility and membrane permeability. To address these issues, ETP is commercially available as soft gelatin capsules containing ETP incorporated into an emulsion (ETP emulsion) consisting of polyethylene glycol (PEG), glycerol, and citric acid anhydrous (Solano et al., 2013). Although the development of ETP emulsion addressed the problems related to ETP solubility, variations in the pharmacokinetic profile persisted (Toffoli et al., 2001). This may have been due to differences in the rate of drug uptake through the gastrointestinal mucosa, resulting from the poor permeability of ETP, or to P-glycoprotein (P-gp)-mediated efflux (Stuurman et al., 2013). Slevin and Joel suggested that a more soluble and readily absorbed preparation in the form of ETP phosphate may overcome this problem (Slevin & Joel, 1993). However, variable conversion of ETP phosphate to ETP within the intestinal lumen after oral administration resulted in poor absorption, and the pharmacokinetic variability appeared unaltered, thus limiting its use (Varsha et al., 2017). In recent years, several approaches such as modified nano-structured lipid carrier preparations, phospholipid complex self-emulsifying drug delivery systems, ETP–rubusoside nanoparticles, ETP-loaded poly(ε-caprolactone) implants, and poly-l-lactic acid-based ETP-loaded implants, have been examined to increase the bioavailability of ETP (Zhang et al., 2011, 2012; Solano et al., 2013; Khalid et al., 2018; Wu et al., 2020). These strategies focused mainly on increasing the oral absorption of ETP by increasing its solubility via physiochemical modifications, incorporation into nanoemulsions, nanoparticle formulation, or maintaining low and sustained release of ETP through the use of implants. On the other hand, increasing the oral absorption of ETP by suppressing P-gp has also been suggested. Mo et al. suggested the use of *N*-octyl-*O*-sulfate chitosan as a formulation excipient to suppress P-gp and increase the oral absorption of ETP (Mo et al., 2011). Moreover, P-gp substrates, such as verapamil, cyclosporine A (Cys A), and quercetin, have been utilized (Bisogno et al., 1998; Piao et al., 2008; Li & Choi, 2009). All of the above approaches resulted in considerable improvements in oral absorption compared with oral ETP suspension (ES). However, few studies have utilized additional intestinal transporter-targeted nanoparticle delivery to enhance the bioavailability of ETP.

**Figure 1.**
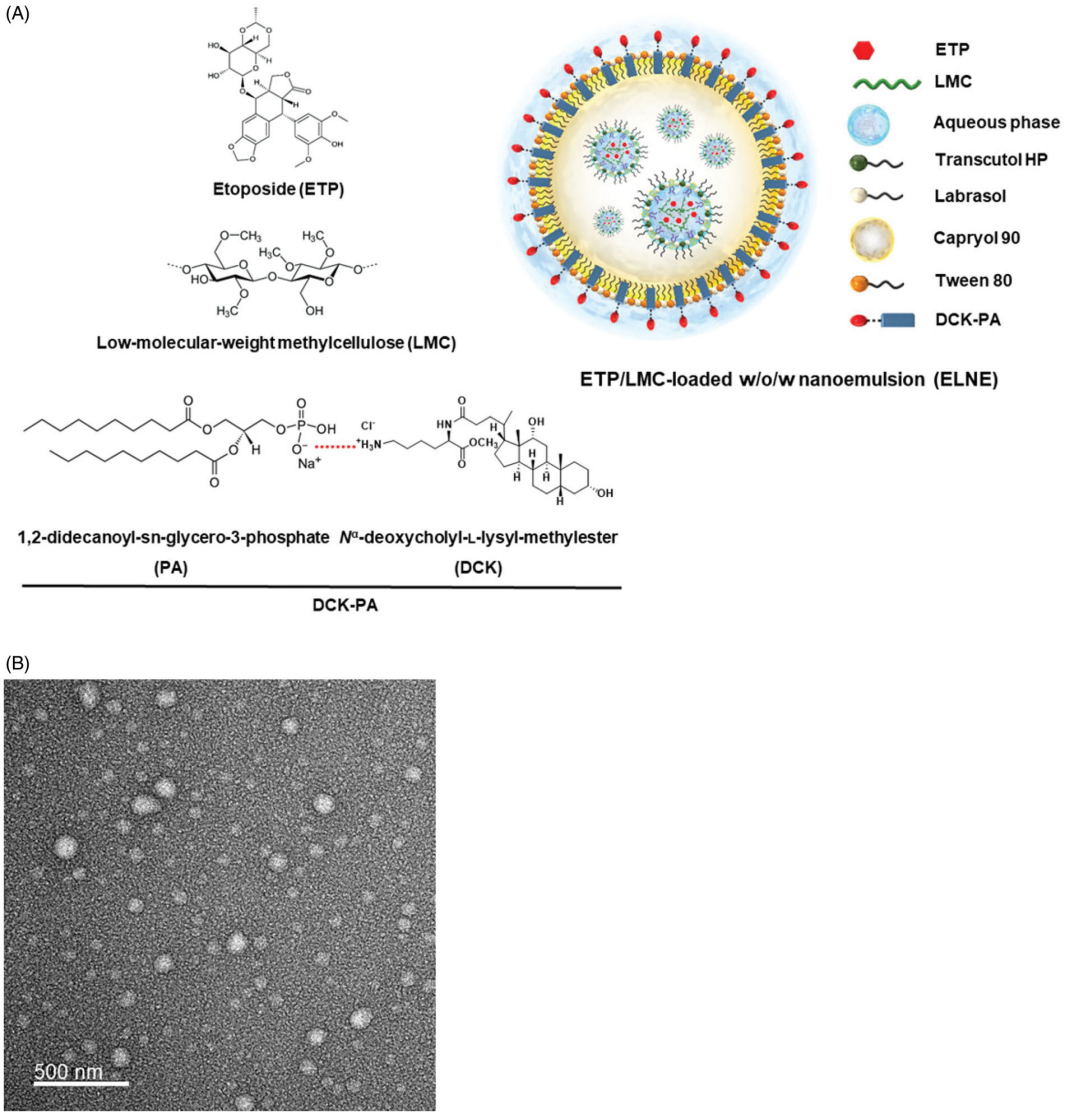
(A) Schematic illustration of an etoposide/low-molecular-weight methylcellulose (ETP/LMC)-loaded water-in-oil-in-water (w/o/w) nanoemulsion incorporating an ionic complex of *N*^α^-deoxycholyl-l-lysyl-methylester and 1,2-didecanoyl-sn-glycero-3-phosphate (sodium salt) (DCK–PA) (ELNE#7). (B) Transmission electron micrographic image of ELNE#7. Scale bar, 100 nm.

The main objective of our investigation was to design a drug delivery system capable of markedly increasing both the aqueous solubility and intestinal permeability of ETP. Multiple approaches were used. First, the aqueous solubility of ETP was increased by forming a physical inclusion complex with low-molecular-weight methylcellulose (LMC) (ETP/LMC). Second, the permeability was enhanced by loading the ETP/LMC complex into a water-in-oil-in-water (w/o/w) multiple nanoemulsion to formulate ETP/LMC-nanoemulsion (ELNE). Third, the oral absorption was further improved by actively targeting an apical sodium-dependent bile acid transporter (ASBT) present in the ileum by anchoring a complex of the cationic bile acid derivative *N*^α^-deoxycholyl-l-lysyl-methylester (DCK) and the anionic lipid 1,2-didecanoyl-sn-glycero-3-phosphate (sodium salt) (PA) (DCK–PA), as a functional ligand, into the oil phase of ELNE (Pangeni et al., 2016; Chung et al., 2018) (Figure 1(A)). Next, to confirm the increases in permeability and oral absorption of ELNE, we evaluated its ability to permeate artificial membranes and Caco-2/HT29-MTX-E12 monolayers. The additional presence of ASBT-mediated transport in combination with various other absorption routes followed by the use of nanoparticles was examined across the Caco-2 monolayer in the presence of specific biochemical inhibitors of distinct cellular uptake pathways. Finally, the *in vitro* cytotoxicity of ELNE in Lewis lung carcinoma (LLC) and A549 cells and the oral bioavailability in rats were evaluated and compared with those of commercially available hydrophilic ETP emulsion.

## 2. Materials and methods

### 2.1. Materials

ETP, teniposide (internal standard for ETP), methylcellulose (molecular weight: 60,000 Da), hexafluro-2-propanol, polyoxyethylene sorbitan monooleate (Tween 80), d-alpha-tocopherol polyethylene glycol succinate (TPGS), sodium carboxymethylcellulose (NaCMC), deoxycholic acid, chlorpromazine, methyl-β-cyclodextrin (MBCD), brefeldin A, genistein, actinomycin D, Cys A, ethylene glycol-bis-(2-aminoethyl ether)-*N*,*N*,*N*′,*N*′-Cys A, ethylene glycol-bis-(2-clofazimine) were obtained from Sigma-Aldrich Inc. (St. Louis, MO). Cellulase (105 U/mg) was purchased from Worthington Biochemical Corporation (Lakewood, NJ). Propylene glycol monocaprylate (Capryol 90), caprylocaproyl macrogol-8 glycerides (Labrasol), diethylene glycol monoethyl ether (Transcutol HP), and Tween 80 were provided by Gattefossé (Saint Priest, France). PA (10:0) was obtained from Avanti Polar Lipids (Alabaster, AL). All other chemicals for high-performance liquid chromatography (HPLC) and liquid chromatography/tandem mass spectrometry (LC/MS) analyses were obtained from Thermo Fisher Scientific Inc. (Waltham, MA).

### 2.2. Animals

Sprague–Dawley rats (male, 6–7 weeks old, 200–250 g) were provided by OrientBio (Gwangju, Republic of Korea). Ethical approval for this study was obtained from the Institutional Animal Care and Use Committee of Mokpo National University (Jeonnam, Republic of Korea) (approval no. MNU-IACUC-2020-006). All animal experiments were performed in accordance with the National Institutes of Health Guidelines for the Care and Use of Laboratory Animals and the guidelines of the Institutional Animal Care and Use Committee.

### 2.3. Preparation and characterization of ELNE

The physical complex ETP/LMC was prepared to enhance the solubility of ETP in water. First, LMC was prepared by enzymatic degradation of methylcellulose, as described previously (Chung et al., 2018). To prepare the ETP/LMC complex, ETP and LMC were dissolved in hexafluro-2-propanol at a concentration of 100 mg/mL with feed weight ratios of ETP to LMC of 1:1, 1:5, and 1:10, respectively. The admixture of ETP and LMC was then evaporated at 37 °C using a rotary evaporator, forming a thin film in a round-bottom flask. Subsequently, the thin film was dispersed in 3 mL distilled water at 50 °C with continuous sonication for 10 minutes. The ETP/LMC solution was then incubated at −4 °C for 30 minutes and filtered through a 0.22-µm polyvinylidene difluoride filter. Finally, the filtered solution was freeze-dried to obtain a powdered form of the ETP/LMC complex.

To improve the intestinal permeability of ETP via bile acid transporter-mediated uptake, the DCK–PA complex was prepared as follows. First, DCK was synthesized by conjugating deoxycholic acid to a positively charged lysine, as described previously (Pangeni et al., 2016). Then, 100 mg PA was dispersed in 20 mL water, vortex mixed, and sonicated for 20 minutes. Subsequently, 114.6 mg DCK was completely dissolved in water and then added gradually in a dropwise manner to the PA dispersion with continuous stirring at a 1:1 molar ratio. The DCK–PA dispersion was freeze-dried at −70 °C to obtain the DCK–PA complex in powder form.

Next, the ETP/LMC complex was incorporated into the multiple w/o/w nanoemulsion, with or without DCK–PA complex. A two-step spontaneous emulsification method was used to prepare w/o/w nanoemulsion based on the pseudo-ternary phase diagram (Supporting Information, Figure S1). First, the primary w/o nanoemulsion was prepared. Briefly, ETP/LMC was dispersed in water followed by oil phase titration using Capryol 90, Labrasol, and Transcutol HP as the aqueous, oil, surfactant, and co-surfactant phases, respectively. The primary nanoemulsion was comprised of a 25.9% (w/w) aqueous solution of ETP/LMC, 68.3% surfactant/co-surfactant mixture (*S*_mix_; 1:2), and 5.80% oil phase (Supporting Information, Table S1). Second, the w/o/w ETP/LMC was prepared by aqueous titration using the primary nanoemulsion, Tween 80, and deionized water as the secondary oil, surfactant, and aqueous phases, respectively. The final composition of w/o/w nanoemulsion was as follows: 70.0% (w/w) w/o nanoemulsion (oil phase), 9.69% (w/w) surfactant, and 19.3% (w/w) deionized water (Supporting Information, Table S1) (Pangeni et al., 2016).

The average droplet size, polydispersity index (PDI), and zeta potential of ELNE were determined using a dynamic laser light scattering analyzer (Malvern Zetasizer Nano ZS90; Malvern Instruments, Malvern, UK). Morphological evaluation of the selected ELNE was performed by transmission electron microscopy (JEM-200; JEOL, Tokyo, Japan).

### 2.4. *In vitro* intestinal membrane permeability of ELNE

#### 2.4.1. *In vitro* artificial intestinal membrane permeability

A parallel artificial membrane permeability assay was performed using pre-coated 96-well plates designed for this assay (Corning, Corning, NY) according to the manufacturer’s instructions. Briefly, 300 µL PBS (pH 6.8) was added to the receiver plate, and 200 µL of each formulation diluted in PBS (pH 6.8) containing 100 µg/mL ETP was added to each well of the filter plate. The plates were then assembled, and the system was incubated at room temperature for five hours without shaking. After five hours, the plates were disassembled, and samples were withdrawn from both the donor and acceptor plates. The concentration of ETP that permeated through the artificial membrane was measured by HPLC at 254 nm. A C18 column (4.6 × 150 mm, 5 µm, 100 Å; 50-µL sample injection) was used with a mobile phase consisting of acetonitrile and water (40:60, v/v) at a flow rate of 1 mL/min. The effective permeability (*P_e_*) of each sample was calculated according to the formula:

(1) $$P_{e}=-\ln[1-\frac{C_{A}(t)}{C_{\mathrm{equilibrium}}}]/[A\;\times (\frac{1}{V_{D}}+\frac{1}{V_{A}})\times t]$$

where *P_e_* is the permeability (cm/s), *A* is the effective permeation area (0.288 cm^2^), *V*_D_ is the volume of the donor well (0.2 mL), *V*_A_ is the volume of the acceptor well (0.3 mL), *t* is the total incubation time in s, *C*_A_(*t*) is the concentration of the drug in the acceptor well at time *t*, and *C*_equilibrium_ represents $\frac{\left[C_{D}(t)\;\times \;V_{D}\;+\;C_{A}(t)\;\times \;V_{A}\right]}{\left(V_{D}\;+\;V_{A}\right)},$ where *C*_D_(*t*) denotes the concentration of the drug in the donor well at time *t*.

#### 2.4.2. *In vitro* permeability across a Caco-2 and HT-29-MTX-E12 monolayer

Caco-2 (ATCC^®^ HTB-37™; American Type Culture Collection, Manassas, VA) and HT29-MTX-E12 (EACC 12040401; Public Health England, Oxford, UK) cells were seeded onto polycarbonate 24-well Transwell^®^ filters at a density of 1 × 10^5^ cells/well (8:2). The confluent monolayers obtained (18–21 days) were utilized to assess the *in vitro* permeability. Culture media in the apical and basolateral compartments were replaced at two-day intervals, and the integrity of the monolayer was monitored by measuring the transepithelial electrical resistance (TEER). Only cell monolayers with a TEER value of 450 ± 100 Ω·cm^2^ were used in the study. Before the experiments, culture medium in both chambers was replaced with pre-warmed Hank’s balanced salt solution (HBSS). Cultures were then stabilized at 37 °C for 20 minutes. For the permeation studies, 0.1 mL drug formulation diluted with HBSS (equivalent to 100 µg/mL ETP) was added to the apical side, and the basolateral side was replaced with 0.6 mL fresh HBSS. The treated cells were incubated at 37 °C ± 0.5 °C. The amount of permeated drug was determined by collecting 0.2 mL samples from the basolateral compartment, followed by replacement with 0.2 mL fresh HBSS at 0, 0.5, 1, 2, 3, 4, and 5 hours. The collected samples were then filtered through 0.45-µm polyvinylidene difluoride filters, and the concentrations of ETP in the samples were determined by HPLC using a UV detector at 254 nm, as described above.

The apparent permeability (*P_app_*) of ETP in various formulations was calculated as follows:

(2) $$P_{\mathrm{app}}=\frac{dQ}{dt}\;\times \frac{1}{A\;\times \;C_{0}}$$

where d*Q*/d*t* is the slope of the cumulative drug permeated versus time (µg/h), *A* is the surface area of the monolayer (0.33 cm^2^), and *C*_0_ is the initial concentration of ETP on the apical side (µg/mL).

### 2.5. Cellular uptake by Caco-2 and ASBT-transfected MDCK cells

Enhanced cellular uptake of ELNE was assessed in Caco-2 cells. Briefly, Caco-2 cells were seeded at 5 × 10^4^ cells/coverslip coated with Cell-Tak adhesive (Corning, Corning, NY) and allowed to proliferate until they had formed a monolayer. Culture medium was replaced with an aqueous solution of ETP/LMC, ETP/LMC in 0.3% NaCMC, ENE, ELNE#1, and ELNE#7 formulations. All samples were co-loaded with coumarin-6 (10 µg/mL) as a model hydrophobic drug, then diluted with DMEM to a concentration of 10 µg/mL ETP. Notably, ETP and ETP/LMC cannot be conjugated directly with fluorescence dye. Subsequently, cells were incubated at 37 °C for three hours and treated with phalloidin-rhodamine (100 nM) to stain actin filaments; the nuclei were counterstained with 4′,6-diamidine-2′-phenylindole dihydrochloride. The cells were rinsed three times with PBS (pH 7.4). Cellular uptake was then evaluated using a confocal laser scanning microscope (Carl Zeiss, Oberkochen, Germany).

After DCK–PA had been incorporated into the nanoemulsions, enhancement of the intestinal permeability of ELNE via interaction with the bile acid transporter was assessed using human ASBT gene-transfected MDCK cells. MDCK cells were seeded onto Cell-Tak-coated coverslips at a density of 1 × 10^4^ cells/coverslip, then transfected with the human ASBT gene using Lipofectamine 2000^®^ (Thermo Fisher Scientific Inc., Waltham, MA). After the transfected cells had formed a confluent monolayer, they were treated with coumarin-6-co-loaded ETP/LMC solution; ETP/LMC in 0.3% NaCMC; ENE; and ELNE#1, #6, and #7 diluted with DMEM (each containing 10 µg/mL coumarin-6 and ETP, respectively). The cells were then incubated at 37 °C for three hours. Subsequently, the cells were fixed in 4% cold paraformaldehyde, blocked in blocking buffer (0.3% Triton X-100 and 10% normal goat serum in PBS, pH 7.4), and incubated with an anti-human ASBT antibody. Further staining with 10 µg/mL Alexa Fluor 546-labeled secondary antibody was performed for one hour, and nuclei were counterstained with 4′,6-diamidino-2′-phenylindole dihydrochloride (1 µg/mL) for five minutes. Finally, fluorescence images were obtained using a confocal laser scanning microscope.

### 2.6. Intestinal transport mechanism of ELNE

Caco-2 and HT29-MTX-E12 cell monolayers were obtained as described above. The experiment was performed once monolayers with a TEER value >350 Ω·cm^2^ had formed. To elucidate the transcellular pathway involved in ELNE absorption, 0.1 mL HBSS containing inhibitors of specific cellular uptake pathways and 0.6 mL HBSS were added to apical and basal chambers, respectively, and preincubated at 37 °C for 30 minutes (Supporting Information, Table S2). Thereafter, the solution in the apical compartment was replaced with 0.1 mL ELNE diluted in HBSS (equivalent to 100 µg/mL ETP) along with the corresponding inhibitors, while the solution in the basolateral compartment was replaced with 0.6 mL fresh HBSS. The cells were then incubated at 37 °C. Aliquots (200 µL) of sample solution were withdrawn from the basolateral compartment of each well at 0, 0.5, 1, 2, 3, 4, and 5 hours and replaced with the same volume of fresh HBSS (Qu et al., 2018).

Furthermore, to explore the role of P-gp-mediated efflux during the permeation of ELNE, a permeability bidirectional transport study of an aqueous solution of ETP/LMC and ELNE#7 was conducted, i.e. in both apical-to-basal (AB; uptake) and basal-to-apical (BA; efflux) directions, in the presence or absence of Cys A (specific inhibitor of P-gp efflux). For AB transport (uptake), 0.1 mL drug solution (equivalent to 200 µg/mL ETP) with Cys A was added to the apical chamber (donor) of the Transwell^®^. Samples (200 μL) were withdrawn from the basolateral chamber (receiver) at defined time points (0, 0.5, 1, 2, 3, 4, and 5 hours after drug loading), followed by replacement with fresh HBSS to maintain the initial volume. For BA transport (efflux), 0.6 mL drug solution (equivalent to 200 µg/mL ETP) without Cys A was added to the basolateral chamber (donor), and samples (0.05 mL) were withdrawn from the apical chamber (receiver) in a manner similar to that described for AB transport.

Next, to evaluate the effect of tight junction opening on ELNE transport, an extracellular Ca^2+^ chelating agent (EGTA) was applied to reversibly open intracellular tight junctions in the Caco-2/HT29-MTX-E12 cell monolayers. Moreover, to investigate the effect of EGTA in combination with all inhibitors except Cys A (specific inhibitor of P-gp efflux) on ELNE transport, Caco-2/HT29-MTX-E12 cell monolayers with a TEER value >350 Ω·cm^2^ were treated with 0.1 mL HBSS (Ca^2+^-free medium) containing 2.5 mM EGTA with or without inhibitors except Cys A. The basolateral side was filled with 0.6 mL HBSS (Ca^2+^-free medium). The Transwell^®^ was then preincubated at 37 °C for 45 minutes, and the integrity of the cell monolayer was monitored to ensure that the TEER value remained ≤70 Ω·cm^2^, thus confirming opening of the junctional complex. Medium in the apical compartment was replaced with 0.1 mL HBSS (with 1.8 mM Ca^2+^) containing ELNE#7 (equivalent to 100 µg/mL ETP) alone or with all inhibitors except Cys A. Next, 0.6 mL HBSS (with 1.8 mM Ca^2+^) was added to the basolateral side of each well, and the cells were incubated at 37 °C for an additional five hours. At predetermined time points, 0.2 mL medium on the basolateral side was withdrawn and replaced with the same volume of fresh HBSS. In addition, the integrity of the monolayer was checked by measuring the TEER value, which returned to same volume^2^ at 2 and 5 hours after drug loading, thus confirming restoration of transmembrane resistance (Artursson & Magnusson, 1990).

### 2.7. *In vitro* cytotoxicity

LLC and A549 cells were seeded in 96-well plates at a density of 1 × 10^4^/well in 100 µL DMEM or RPMI 1640 containing 10% FBS, respectively. After incubation at 37 °C for 24 hours, the cells were treated with a series of doses of an aqueous dispersion of ETP, ETP/LMC solution, ETP solution constituted in a mixture of citric acid (0.2%), benzyl alcohol (3.06%), Tween 80 (8.16%), PEG 400 (66.33%), and ethanol (22.24%) (ETP-IV), ETP emulsion, or ELNE#7 diluted in DMEM or RPMI 1640 with 1% FBS. The cells were incubated for 24 hours. To determine cell viability, 10 μL 2-(2-methoxy-4-nitrophenyl)-3-(4-nitrophenyl)-5-(2,4-disulfophenyl)-2H-tetrazolium monosodium salt (WST-8) solution diluted in 100 µL DMEM or RPMI 1640 was added to each well. The cells were incubated for two hours, and the absorbance at 450 nm was measured using a microplate reader. Untreated cells were used as controls (100% viability). Wells without WST-8 were used as blanks to calibrate the spectrophotometer to zero absorbance. The percentage of viable cells was calculated by comparing the values of the treated cells with those of the untreated cells.

### 2.8. *In vivo* pharmacokinetic rat study

To evaluate the beneficial effect of ETP/LMC complex formation on the intestinal absorption of ETP, as well as its effect on incorporation into ELNE, 400 µL ETP dispersed in water (20 mg/kg ETP), ETP in 5% DMSO (20 mg/kg ETP), ETP/LMC solution (20 mg/kg ETP), ETP emulsion (20 mg/kg ETP), and ELNE#7 (20 mg/kg ETP) were orally administered to rats. In addition, 200 µL ETP-IV (20 mg/kg ETP) was injected via the tail vein to evaluate oral bioavailability. Next, blood samples (150 µL) were collected into heparinized tubes from the retroorbital plexus at predetermined time points, then mixed with 50 µL 3.8% sodium citrate solution and immediately centrifuged (2500×*g*, 15 minutes, 4 °C). Plasma samples were separated and kept frozen at −70 °C until the analysis. LC/MS was performed to determine the concentration of ETP in plasma. Briefly, 100 µL plasma was spiked with 100 µL internal standard (2.5 µg/mL teniposide). Next, 100 µL acetonitrile was added to the plasma spiked with internal standard. Extraction was performed using 3 mL *tert*-butyl methyl ether/*n*-hexane (90:10, v/v) by vortex mixing and centrifugation at 10,000 rpm for five minutes at 4 °C. After the mixture had been centrifuged, the organic phase was transferred to another vial and then evaporated to dryness using a rotatory evaporator at a reduced boiling point. Finally, residues were reconstituted in 100 µL acetonitrile and subjected to LC/MS using the Agilent 6120 Quadruple LC/MS system with a Luna C18 column (150 × 4.6 mm, 5 µm) as the stationary phase. Aliquots (20 µL) from each sample were eluted in the mobile phase, consisting of acetonitrile (50%) with a 50% isocratic gradient of deionized water containing 0.1% (v/v) formic acid, for 15 minutes at a flow rate of 0.5 mL/min. ETP and internal standard were ionized using an electrospray ionization source in positive-ion mode under the following source conditions: capillary voltage, 4 kV; drying gas flow rate, 10.0 L/min; drying gas temperature, 300 °C. Quantitative analyses of protonated molecular ions were performed at ([M + Na]^+^=589.3) and ([M + Na]^+^=675.2) for ETP and teniposide, respectively (Wang et al., 2009).

### 2.9. Pharmacokinetic and statistical analyses

Pharmacokinetic parameters were assessed using a non-compartmental method in WinNonlin^®^ software (ver. 5.3; Pharsight Corporation, Mountain View, CA). One-way analysis of variance followed by Tukey’s multiple-comparison test was used to compare more than two mean values. All data are expressed as means ± standard deviation. In all analyses, *p*<.05 was taken to indicate statistical significance.

## 3. Results and discussion

### 3.1. Preparation and characterization of ELNE

To enhance the aqueous solubility, loading ability, and stability of ETP in primary nanoemulsion, physical complexes between ETP and LMC (ETP/LMC) with different weight ratios (1:1, 1:5, and 1:10) were formed, and their aqueous solubilities and artificial membrane permeabilities were evaluated. Based on the highest aqueous solubility and artificial membrane permeability, ETP/LMC (1:5) was selected for loading into the nanoemulsion (Supporting Information, Table S3). Next, ETP/LMC (1:5) was loaded into the inner aqueous phase of the optimum w/o/w nanoemulsion based on the pseudo-ternary phase diagram, thereby yielding ELNE (Supporting Information, Figure S1). Moreover, successful incorporation of ETP/LMC (1:5) into nanoemulsion was confirmed by analysis of particle size, PDI, and zeta potential. ELNE#1 showed a particle size and PDI of 189 ± 10.3 nm and 0.377 ± 0.015, respectively, which confirmed the formation of an ETP/LMC-incorporated nanoemulsion (Supporting Information, Table S4). To further increase the permeability of ELNE via ASBT-mediated transport, DCK–PA was anchored into ELNE#1. The formation of an ionic complex between DCK and PA was confirmed by comparing the individual surface charges of PA, DCK, and DCK–PA. The zeta potential of PA was −83.4 ± 3.87 mV, and that of DCK was 29.4 ± 0.57 mV. However, after the formation of DCK–PA, the surface charge of the complex was −28.0 ± 0.21 mV. Therefore, the increase in surface charge of PA in DCK–PA micelles indicated mitigation of its negative charge by equimolar cationic DCK counterions, which supported formation of the DCK–PA complex. Next, to confirm the successful anchorage of the DCK–PA complex into the nanoemulsion, we incorporated PA into the ELNE#1. As expected, the surface charge of ELNE#1 decreased from −14.6 ± 1.68 mV to −47.4 ± 1.95 mV in PA-ELNE#7, whereas it was −11.1 ± 0.915 mV in DCK–PA-incorporated ELNE#1, i.e. ELNE#7, respectively. These findings indicate the successful anchorage of DCK–PA into the ELNE and confirmed that the hydrophobic tail component of PA was well adsorbed into the oil phase, whereas the anionic head remained exposed toward the o/w interphase.

The optimal ELNE (ELNE#7) had a droplet size, PDI, and zeta potential of 172 ± 5.70 nm, 0.290 ± 0.005, and −11.1 ± 0.915 mV, respectively. The morphology and surface structure of ELNE#7, as determined by TEM, were also suggestive of nano-sized droplets based on the results of particle size analysis (Figure 1(B)). These results further indicated that *S*_mix_, by reducing the interfacial energy, induced thermodynamic stability of the nanoemulsion.

### 3.2. *In vitro* permeability study of ELNE

To enhance the aqueous solubility, we first complexed ETP with LMC, thereby forming water-soluble ETP/LMC. As expected, the use of an ETP/LMC complex improved the ETP solubility in a manner that increased linearly up to ETP/LMC (1:10) (Supporting Information, Table S3). However, the *P_e_* of ETP/LMC (1:10) was 201% lower than that of ETP/LMC (1:5). Thus, the enhancement of ETP/LMC (1:10) permeability did not appear to coincide with enhancement of solubility. The concomitant reduction in permeability of ETP/LMC with the proportional enhancement of solubility may have occurred because of the diminished membrane/aqueous partition, which resulted in a solubility–permeability tradeoff (Beig et al., 2015). In addition, the poorly soluble ETP dispersion showed minimal diffusion across the artificial membrane. However, the ETP/LMC solution alone and in 0.3% NaCMC showed 4.78- and 6.92-fold enhancements of *P_e_*, compared with ETP in 5% DMSO, respectively (Supporting Information, Table S1 and Table 1). Therefore, based on its improved solubility and permeability compared with ETP, an ETP/LMC complex with a weight ratio of 1:5 was formulated into the nanoemulsion to achieve maximum enhancement of its absorption (Chung et al., 2018). To further improve the permeability of ETP, we incorporated the ETP/LMC complex into the w/o/w nanoemulsion (ELNE). As expected, ELNE#1 exhibited 4.91-fold greater *P_e_*, compared with that of ETP/LMC solution (Table 1). The substantial improvement in permeability exhibited by ELNE#1 could have resulted from the increased membrane/aqueous partition coefficient of ETP/LMC-incorporated nanoemulsive oil droplets. In contrast, *P_e_* values of ETP-incorporated nanoemulsion (ENE) and ETP emulsion were 165% and 326% lower than those values for ELNE#1, respectively (Table 1). This revealed an advantageous effect of ETP/LMC, compared with ETP, following incorporation into nanoemulsive droplets. Next, to further enhance the permeability of ELNE#1, TPGS was incorporated into ELNE#2, #3, and #4. TPGS-incorporated ELNE#2 and #3 did not show any further improvement in permeability, compared with ELNE#1. Furthermore, the *P_e_* of ELNE#4 decreased by 1.54-fold, compared with that of ELNE#1 (Table 1). This result showed that across an artificial membrane, the solubility–permeability exhibited a tradeoff when TPGS was used as a surfactant (Beig et al., 2017). In addition, the DCK–PA complex was also incorporated into the nanoemulsion as a recognition moiety for ASBT-facilitated uptake (ELNE#5, #6, and #7). Its effect on *P_e_* was then monitored: the *P_e_* of ELNE#5 and #6 showed trivial enhancements, whereas the *P_e_* of ELNE#7 showed a 1.24-fold enhancement, compared with ELNE#1 (Table 1). The considerable increase in *P_e_* of ELNE#7 could be attributed to the DCK constituent of bile acid (i.e. deoxycholic acid), which enhanced *P_e_* via its well-known solubility- and permeability-improving properties (Kim et al., 2011).

**Table 1.** Effective and apparent permeabilities of ETP, ETP/LMC, ETP emulsion, ENE, and ELNE formulations.

Formulation	Effective permeability (*P_e_*, ×10^–6^ cm/s)	Apparent permeability (*P_app_*, ×10^–6^ cm/s)
ETP dispersed in water	0.439 ± 0.079	1.86 ± 0.736
ETP/LMC solution	1.85 ± 0.920	4.14 ± 0.619
ETP dispersed in 0.3% NaCMC	1.61 ± 0.180	3.73 ± 0.265
ETP/LMC in 0.3% NaCMC	2.68 ± 0.252	4.32 ± 0.742
ETP in 5% DMSO	0.387 ± 0.091	2.92 ± 0.271
ENE	5.50 ± 1.41	4.82 ± 0.586
ETP emulsion	2.79 ± 0.487	4.25 ± 0.926
ELNE#1	9.10 ± 0.407	6.42 ± 1.58
ELNE#2	9.50 ± 0.393	6.63 ± 1.76
ELNE#3	9.39 ± 1.220	6.76 ± 0.396
ELNE#4	5.90 ± 0.120	6.26 ± 1.03
ELNE#5	9.35 ± 0.482	6.79 ± 0.408
ELNE#6	10.1 ± 0.303	7.93 ± 1.17
ELNE#7	11.4 ± 0.267	9.60 ± 1.10

Each value represents the mean ± standard deviation (*n*= 6).

Next, the *in vitro P_app_* of different formulations described in the Supporting Information, Table S1 were examined. These data are presented in Table 1. The *P_app_* values of ETP/LMC solution alone and in 0.3% NaCMC were enhanced by 141% and 147%, compared with ETP in 5% DMSO, respectively. These findings could be attributed to the improved aqueous solubility and permeability of ETP in the ETP/LMC inclusion complex. To further enhance the *P_app_* of ETP/LMC, it was incorporated into the w/o/w nanoemulsion. Notably, the *P_app_* of ELNE#1 was enhanced by 2.19- and 1.51-fold, compared with ETP in 5% DMSO and ETP emulsion alone, respectively. The further improvement in permeability exhibited by ELNE#1 may have been due to alteration of epithelial barrier properties caused by surfactants/co-surfactants, prevention of recognition by P-gp, and facilitation of paracellular transport by Labrasol via disruption of tight junctions at the molecular level (Sha et al., 2005). To further enhance the permeability of ELNE, TPGS was incorporated into ELNE#2, #3, and #4. The addition of TPGS did not result in further improvements in the permeabilities of ELNE#2, #3, or #4, in comparison with ELNE#1. To utilize carrier-mediated transport to increase the oral absorption of ETP, the DCK–PA complex was anchored into the w/o/w nanoemulsion, ELNE#5, #6, and #7, such that relatively lipophilic PA was adsorbed into the oil phase. Amphiphilic DCK remained localized at the o/w interphase and was thus capable of targeting ASBT. The DCK–PA-anchored ELNE#5, #6, and #7 showed 1.05-, 1.23-, and 1.49-fold enhancements of *P_app_*, compared with ELNE#1. The 149% permeability enhancement exhibited by ELNE#7, compared with ELNE#1, suggested the possibility of additional ASBT-mediated transport.

Altogether, the considerable improvement in permeability exhibited by ELNE#7 could be attributed to the combined synergistic solubility and permeability enhancing effects of ETP/LMC-loaded nanoemulsive droplets, as well as ASBT-mediated transport due to the incorporation of DCK–PA as a recognition moiety into the nanoemulsion.

### 3.3. Uptake of the ETP/LMC complex by Caco-2 and ASBT-transfected MDCK cells

**Figure 2.**
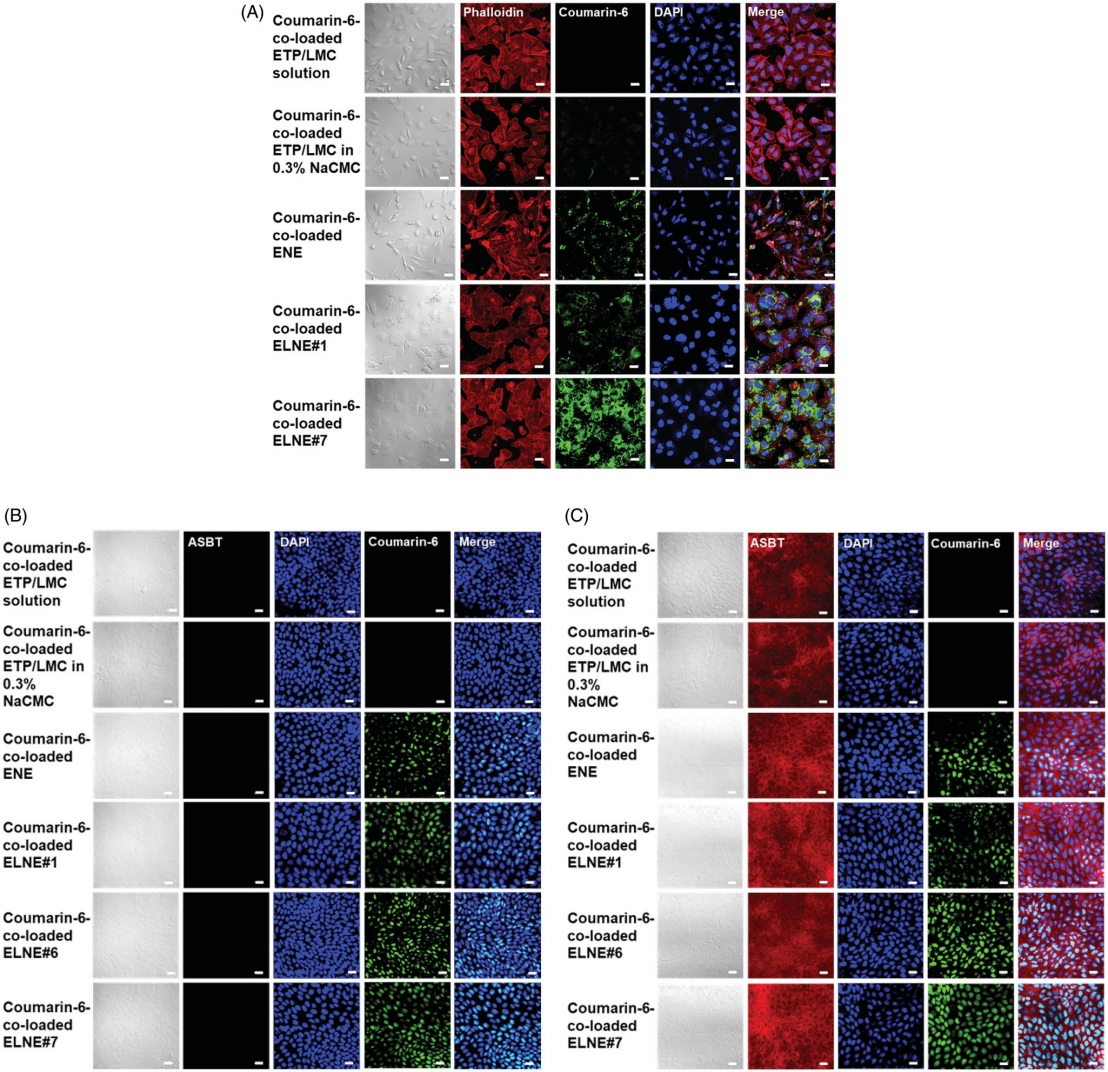
Confocal laser scanning microscopic images of the cellular uptake of different coumarin-6-co-loaded etoposide (ETP) vehicles. (A) Cellular uptake of coumarin-6-co-loaded ETP/LMC solution, coumarin-6-co-loaded ETP/LMC in 0.3% NaCMC, coumarin-6-loaded ENE and ELNE#1, and coumarin-6-loaded ELNE incorporating an ionic complex of *N*^α^-deoxycholyl-l-lysyl-methylester and 1,2-didecanoyl-sn-glycero-3-phosphate (sodium salt) (ELNE#7) by Caco-2 cells. Cellular uptake of coumarin-6-co-loaded ETP/LMC solution, coumarin-6-co-loaded ETP/LMC in 0.3% NaCMC, coumarin-6-loaded ENE and ELNE#1, and coumarin-6-loaded ELNE incorporating an ionic complex of *N*^α^-deoxycholyl-l-lysyl-methylester and 1,2-didecanoyl-sn-glycero-3-phosphate (sodium salt) (ELNE#6 and #7) by (B) MDCK cells or (C) ASBT-transfected MDCK cells. Scale bar, 20 µm.

Intracellular uptake of the ETP/LMC complex-loaded nanoemulsion across the plasma membrane of Caco-2 cells was evaluated qualitatively using confocal laser scanning microscopy. The results of the *in vitro* permeability assays were consistent with the findings of confocal imaging. Coumarin-6-incorporated ENE and ELNE#1 showed greater uptake and assembly of nanoemulsion at the nucleus compared with coumarin-6-dispersed ETP/LMC in water and 0.3% NaCMC, respectively (Figure 2(A)). The enhanced intracellular delivery of coumarin-6 in ENE and ELNE#1 could be attributed to its enhanced aqueous solubility; impact on membrane fluidity and alteration of the epithelial barrier properties by surfactants, such as Labrasol, Transcutol HP, and Tween 80; probable P-gp suppressant effects of Capryol 90, Transcutol HP, and Tween 80; and the presence of additional uptake mechanisms, such as receptor-mediated endocytosis and/or macropinocytosis. In addition, after DCK–PA had been incorporated into the ELNE, considerable enhancement of the permeability of coumarin-6 was observed in ELNE#7 compared with ENE and ELNE#1. The enhanced uptake could be due to the involvement of ASBT-mediated transport during permeation.

Next, to confirm ASBT-mediated uptake after incorporation of DCK–PA into ELNE, ASBT-expressing/non-expressing MDCK cells were utilized for qualitative examination of the uptake of coumarin-6-loaded nanoemulsion droplets of ELNE#6 and ELNE#7. ENE and ELNE#1 showed similar uptake of coumarin-6 in both ASBT-expressing and non-expressing cells. Conversely, confocal images of both ELNE#6 and ELNE#7 showed higher levels of coumarin-6 uptake in ASBT-expressing cells than in non-expressing cells (Figure 2(B,C)). In addition, ELNE#7 showed higher uptake than ELNE#6 in ASBT-expressing cells, suggesting a concentration-dependent effect of DCK–PA with respect to the utilization of ASBT-mediated transport.

Based on the results of *in vitro* permeability and cellular uptake experiments, ELNE#7 was selected as the optimized formulation, i.e. that with the highest cellular uptake and permeability. Hence, further studies (i.e. on the intestinal transport mechanism, *in vitro* cytotoxicity, and oral pharmacokinetics) were performed using ELNE#7.

### 3.4. Intestinal transport mechanism of ELNE

**Figure 3.**
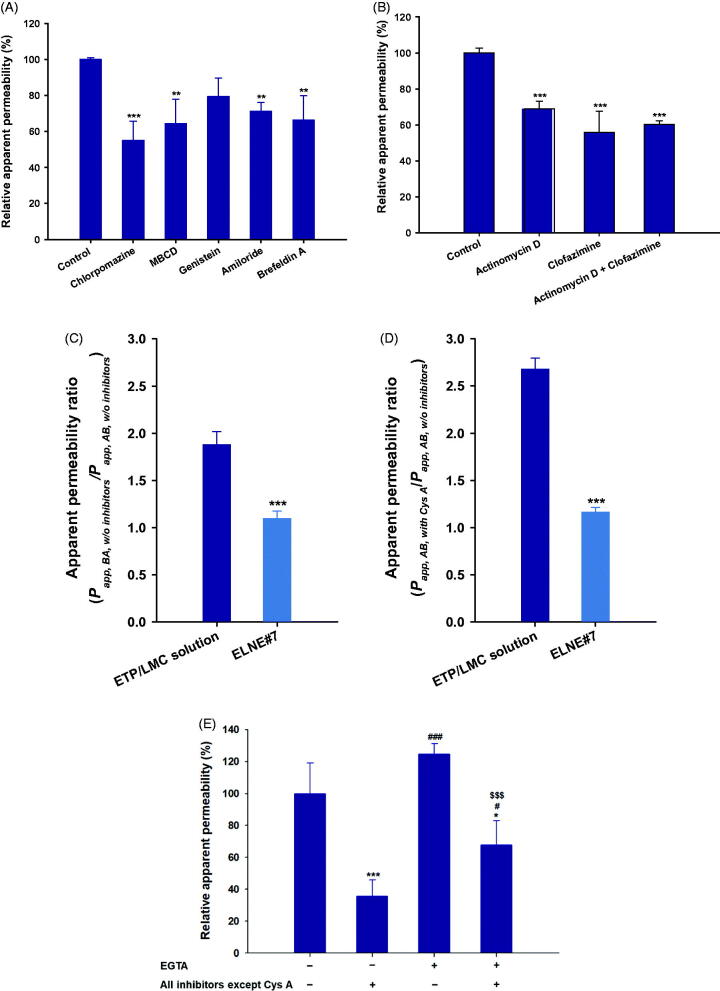
Relative apparent permeability (*P_app_*) values of ELNE#7 without inhibitors (control) and in the presence of various biochemical inhibitors of specific pathways across Caco-2/HT-29-MTX-E12 monolayers. (A) *P_app_* of ELNE#7 across Caco2/HT-29-MTX-E12 monolayers after incubation with various transport inhibitors. (B) *P_app_* of ELNE#7 in the presence of actinomycin D, clofazimine, or both (actinomycin D + clofazimine). Each value represents the mean ± standard deviation (*n*= 4). **p<.01, ***p<.001 compared with the *P_app_* of ELNE#7 in the absence of all inhibitors (untreated control). (C) *P_app_* ratio of ETP/LMC in water and ELNE#7 in both apical to basolateral (AB) and basolateral to apical (BA) directions in the absence of all inhibitors (net efflux ratio; *P_app, BA, w/o inhibitors_*/*P_app, AB, w/o inhibitors_*). (D) Relative *P_app_* ratios of ELNE#7 in the presence of cyclosporine A (Cys A) (net *P_app_* ratio; *P_app, AB, with Cys A_*/*P_app, AB, w/o inhibitors_*). Each value represents the mean ± standard deviation (*n*= 4). ***p<.001 compared with the *P_app_* of ETP/LMC solution. (E) *P_app_* of ELNE#7 with or without EGTA in the presence or absence of all inhibitors except Cys A. Each value represents the mean ± standard deviation (*n*= 4). *p<.05, ***p<.001 compared with the *P_app_* of ELNE#7 without EGTA in the absence of all inhibitors (untreated control); ^#^*p*<.05, ^###^*p*<.001 compared with the *P_app_* of ELNE#7 without EGTA in the presence of all inhibitors except Cys A; ^$$$^*p*<.001 compared with the *P_app_* of ELNE#7 with EGTA in the absence of all inhibitors except Cys A.

To investigate the various transcellular transport routes utilized by ELNE, several biochemical inhibitors specific for the particular mechanism were utilized (Supporting Information, Table S2) (He et al., 2013). After treatment of monolayers with chlorpromazine, an inhibitor of clathrin-mediated endocytosis, the *P_app_* of ELNE#7 decreased by 45.0% compared with the control (without inhibitors). Similarly, inhibitors of caveola-mediated endocytosis (MBCD and genistein) reduced the uptake of ELNE#7 by 35.7% and 21.0%, respectively, compared with the control (without inhibitors) (Figure 3(A)). Moreover, an inhibitor of macropinocytosis, amiloride, reduced the *P_app_* of ELNE#7 by 28.8% compared with the control (without inhibitors) (Figure 3(A)). The considerable reductions in uptake due to treatment with inhibitors of endocytosis and macropinocytosis pathways suggested substantial contributions of these routes to enhancement of ELNE#7 permeation.

In addition, the endoplasmic reticulum (ER) and Golgi complex are important regulators of the secretory ER/Golgi and endocytosis recycling pathways (Burd, 2011). To confirm the involvement of the ER/Golgi pathway during endosomal trafficking of ELNE#7, the effects of a strong inhibitor of this pathway (brefeldin A) were evaluated (Klausner et al., 1992). Notably, brefeldin A reduced the *P_app_* of ELNE#7 by 33.8%, compared with the control (without inhibitors). This finding confirmed considerable lysosomal escape of ELNE#7, and demonstrated the roles of endocytosis and macropinocytosis, respectively (Figure 3(A)).

The structural arrangement of the DCK–PA complex in the ELNE formulation promoted the interaction between DCK and ASBT, thus enhancing absorption via ASBT-mediated transport (Al-Hilal et al., 2014; Park et al., 2017). Therefore, to confirm the involvement of ASBT-mediated transport in the uptake of ELNE#7, specific inhibitors of ASBT and heteromeric organic solute transporter (OST) α/β (actinomycin D and clofazimine, respectively) were utilized. Their effects on the *P_app_* of ELNE#7 were compared with those on the *P_app_* of the control (without inhibitors) (Kanda et al., 1998; van de Wiel et al., 2018). Actinomycin D reduced the *P_app_* of ELNE#7 by 31.1% compared with the control (without inhibitors) (Figure 3(B)). After ASBT-mediated entry into the cytosolic compartment, bile acids bind to the ileal bile acid-binding protein and are exported across the basolateral membrane into the portal circulation by OSTα/β (Dawson & Karpen, 2015). The specific inhibitor of OSTα/β (clofazimine) reduced the *P_app_* of ELNE#7 by 44.1% compared with that of the control (without inhibitors) (Figure 3(B)). Similarly, a combination of inhibitors of both ASBT and OSTα/β resulted in a 39.7% reduction in the *P_app_* of ELNE#7 relative to the control (without inhibitors) (Figure 3(B)). Taken together, these results confirmed the involvement of ASBT-mediated transport, whereby ELNE interacts with ASBT via DCK to cross the plasma membrane and then undergoes translocation in the cytosolic environment by ileal bile acid-binding protein-directed intracellular trafficking, finally exiting through the basolateral membrane of enterocytes via OSTα/β.

Notably, the low and variable bioavailability of ETP is attributed to P-gp efflux. Therefore, to understand the effects of P-gp-mediated efflux during intestinal permeation of ELNE#7, a bidirectional transport study was performed (i.e. AB and BA directions) in the presence of the prominent P-gp inhibitor, Cys A (Anglicheau et al., 2006). The net *P_app_* ratio (i.e. *P_app, AB, with CysA_*/*P_app, AB, w/o inhibitors_*) can be used to elucidate the role of P-gp in the absorptive direction. Furthermore, the net efflux ratio (i.e. *P_app, BA, w/o inhibitors_*/*P_app, AB, w/o inhibitors_*) was measured to evaluate the effects of P-gp during secretory (BA) transport. The P-gp-mediated efflux was considered substantial if the net efflux ratio (*P_app, BA, w/o inhibitors_*/*P_app, AB, w/o inhibitors_*) and net *P_app_* ratio (i.e. *P_app, AB, with CysA_*/*P_app, AB, w/o inhibitors_*) were >2 and >1, respectively. The ETP/LMC solution exhibited moderate efflux across the monolayer, with a net efflux ratio of 1.88. Similarly, the net *P_app_* ratio of ETP/LMC solution increased by 269% in the presence of Cys A (Figure 3(C,D)). These results confirmed the involvement of P-gp-mediated efflux during the transport of ETP/LMC across the intestinal epithelium. Conversely, ELNE#7 showed net efflux ratio and net *P_app_* ratio values of 1.09 and 1.16, respectively, which were both close to 1 (Figure 3(C,D)). These results indicated that Cys A was incapable of further increasing the permeation of ELNE#7. This may have occurred because of the presence of Capryol 90 and Tween 80, which are considered inhibitors of P-gp, or failure to recognize ETP by the P-gp efflux transporter due to the encapsulation of ETP/LMC inside the nanoemulsion droplets (Cornaire et al., 2000). Alternatively, due to the marked increase in the solubility of ETP in ELNE#7, P-gp may have been saturated. Therefore, despite the presence of the P-gp substrate, Cys A, there was no significant impact on the absorption of ELNE#7 (Lin & Yamazaki, 2003).

To evaluate the roles of paracellular transport and passive diffusion, the effects of EGTA – a calcium-chelating agent that binds to free extracellular Ca^2+^ ions and reversibly opens intracellular tight junctions in the Caco-2 monolayer – on the *P_app_* of ELNE were examined (Artursson & Magnusson, 1990). As shown in Figure 3(E), when the monolayer (without EGTA) was treated with all inhibitors except Cys A, the *P_app_* of ELNE#7 was reduced by 64.3% compared with the control (without inhibitors). This finding indicated that 35.7% of ELNE#7 may have entered by passive diffusion or the paracellular route (Figure 3(E)). Notably, the *P_app_* after opening the intracellular tight junctions in EGTA-treated monolayers (treated with all inhibitors except Cys A) was 1.89-fold greater than the *P_app_* of EGTA-untreated monolayers (treated with all inhibitors except Cys A) (Figure 3(E)). These results clearly demonstrated that the opening of intracellular tight junctions could be beneficial for enhancing the *P_app_* of ELNE#7 across the intestinal epithelium. The presence of excipients (e.g. Labrasol) and bile acids, which open tight junctions, in ELNE#7 may have favored its permeation via the paracellular route.

However, the transport mechanisms in this study implicate only transport pathways that can be followed by intact ETP/LMC-incorporated nanoemulsive vehicles; after oral administration, ETP in ELNE#7 can be absorbed via various other mechanisms. After the lipolysis of ELNE, the possible fates of the formulation can be summarized as follows. First, lipolysis or digestion of oil/lipid-based surfactants by gastric and pancreatic lipases can lead to the nanoprecipitation or partitioning of drug out of the oil phase into the surrounding aqueous environment. Second, byproducts of digested oil/lipid-based surfactants, combined with preexisting bile salts, can incorporate the precipitated drug in colloidal micelles and facilitate its uptake. In ELNE#7, we incorporated ETP/LMC into the nanoemulsive system; hence, despite lipolysis, the ETP/LMC can remain solubilized and reincorporated along with digested triglycerides, monoglycerides, fatty acids, lysophosphatidylcholine, and endogenous bile salts. It thus forms a series of colloidal structures, including micelles and uni-/multilamellar vesicles, in gastrointestinal fluid. These micelles will be absorbed by enterocytes, where they can be converted into chylomicron upon re-esterification and presumably reach the systemic circulation through the lymphatic pathway (Ye et al., 2019). Therefore, further studies using an *in vitro* lipolysis model and *in situ* intestinal perfusion assay are necessary for more comprehensive understanding of the enhanced transport of ELNE#7.

### 3.5. *In vitro* cytotoxicity studies

**Figure 4.**
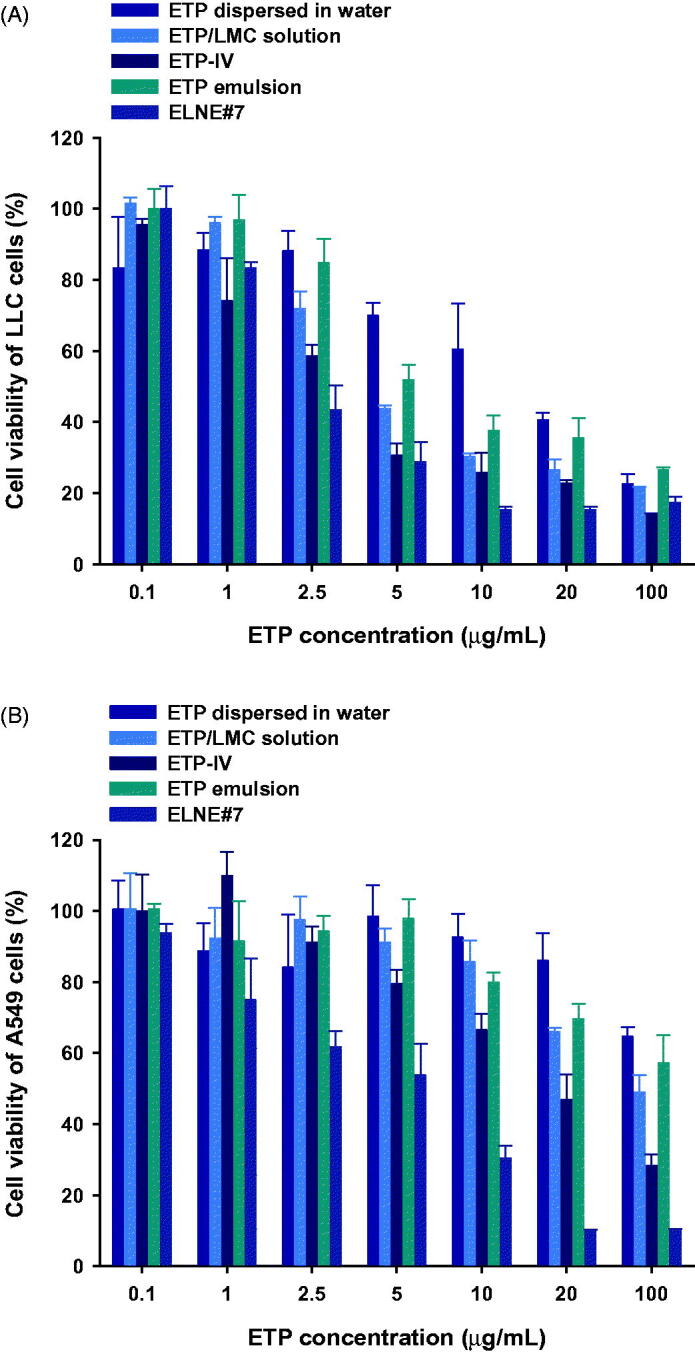
*In vitro* cytotoxic effects of ETP dispersed in water, ETP/LMC solution, ETP-IV, ETP emulsion, and ELNE#7 on (A) LLC and (B) A549 cells after incubation for 24 hours. Each value represents the mean ± standard deviation (*n*= 4).

To evaluate the cytotoxic effects of ELNE#7, its ability to reduce cell viability was compared with those of ETP-IV, ETP emulsion, ETP/LMC solution, and aqueous dispersion of ETP in LLC and A549 cells. The IC_50_ values of ELNE#7, ETP-IV, ETP emulsion, ETP/LMC solution, and ETP dispersed in water were 2.97, 4.09, 4.33, 5.56, and 12.2 µg/mL, respectively, in LLC cells and 5.05, 18.4, 30.0, 29.4, and 118 µg/mL, respectively, in A549 cells (Figure 4). The enhanced cytotoxicity of ELNE#7 compared with ETP-IV and ETP emulsion suggested significant improvements in the aqueous solubility and permeability of ETP/LMC in nanoemulsion droplets, which indeed resulted in marked enhancement of the cytotoxic effects of ELNE#7. Moreover, excipients, such as Capryol 90, Transcutol HP, and Tween 80, could have contributed to the increase in toxicity of ELNE#7 by suppressing the efflux mediated by P-gp, which is generally overexpressed in neoplastic cells, such as A549 and LLC cells. Taken together, the IC_50_ values in both cell lines were within the lower range of the ETP concentration and suggested potent cytotoxic effects of ELNE#7, indicating that it is a better delivery system than ETP emulsion.

### 3.6. *In vivo* pharmacokinetics of ELNE

**Figure 5.**
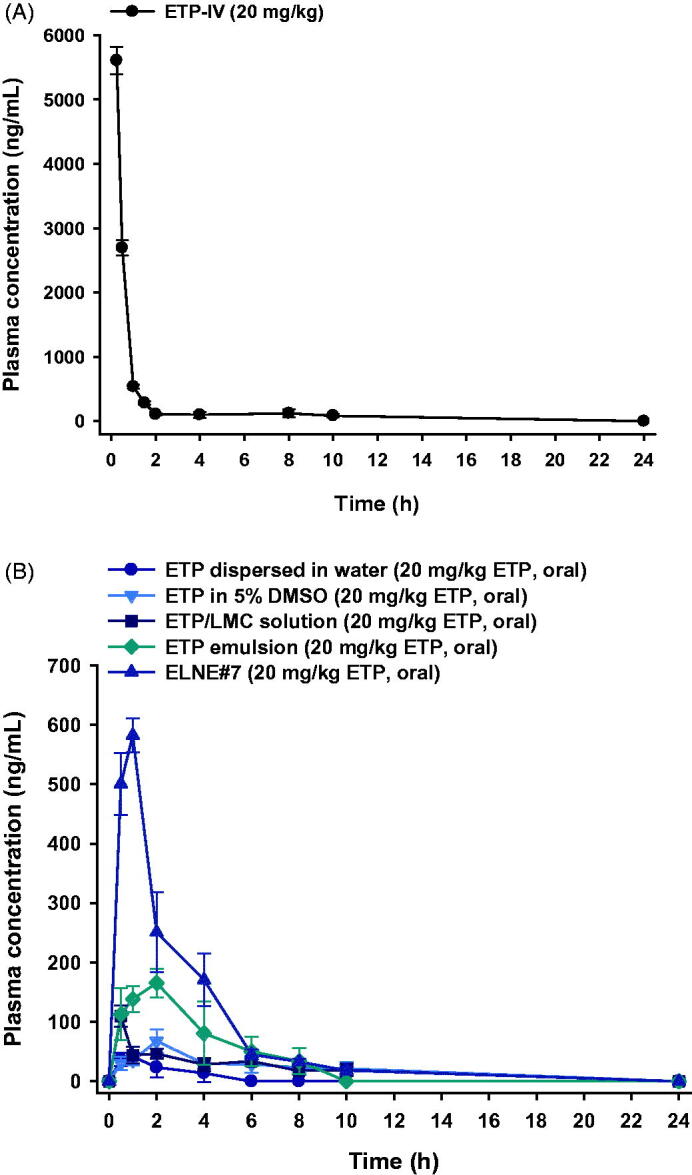
Mean plasma concentration–time profiles of etoposide (ETP) in rats after (A) a single intravenous (IV) administration of 20 mg/kg ETP (ETP-IV) and (B) oral administration of an aqueous suspension of 20 mg/kg ETP (ETP dispersed in water); 5% DMSO solution of 20 mg/kg ETP (ETP in 5% DMSO); aqueous solution of 20 mg/kg ETP/LMC (ETP/LMC solution); ETP-incorporating emulsion (ETP emulsion); and ETP/LMC-loaded nanoemulsion (ELNE) incorporating an ionic complex of *N*^α^-deoxycholyl-l-lysyl-methylester and 1,2-didecanoyl-sn-glycero-3-phosphate (sodium salt) (DCK–PA) (ELNE#7) as 20 mg/kg ETP. Each value represents the mean ± standard deviation (*n*= 4 for each group).

The plasma concentration–time profiles of ETP dispersed in water, ETP in 5% DMSO, ETP/LMC solution, ETP emulsion, and ELNE#7 are shown in Figure 5. The calculated pharmacokinetic parameters are summarized in the Supporting Information, Table S5. The plasma concentrations of ETP/LMC solution, ETP emulsion, and ELNE#7 were significantly higher than that of free ETP. The maximum plasma concentration (*C*_max_) and area under the plasma concentration–time curve (0–24 hours) (AUC_last_) were 2.55- and 2.23-fold greater, respectively, after oral administration of ETP emulsion compared with ETP in 5% DMSO. These changes led to enhancement of oral bioavailability, by 347%. Moreover, the *C*_max_ and AUC_last_ values of ETP emulsion showed 1.57- and 2.09-fold enhancements, respectively, compared with ETP/LMC solution. Notably, the *C*_max_ and AUC_last_ values of ELNE#7 were 3.36- and 2.23-fold higher than the respective values of ETP emulsion. Therefore, the oral bioavailability of ELNE#7 was 224% and 468% greater than the corresponding values of ETP emulsion and ETP/LMC solution. In addition, the times to reach the maximum plasma concentration of ETP dispersed in water, ETP/LMC solution, ETP emulsion, and ELNE#7 were 0.88 ± 0.25, 0.50 ± 0.00, 2.25 ± 1.26, and 1.00 ± 0.00 hours, respectively. The enhanced oral bioavailability of ELNE#7 was consistent with the results of the *in vitro* permeability and mechanistic studies. The increased *P_e_* and *P_app_* values of ELNE#7 across the artificial membrane and Caco-2 monolayer *in vitro*, compared with ETP in 5% DMSO, ETP dispersed in water, ETP/LMC solution, ETP emulsion, and ELNE#1, suggested the involvement of passive diffusion and various other transcellular transport mechanisms in the enhanced oral bioavailability of ELNE#7 (Supporting Information, Table S5). Furthermore, the significant reductions in *P_app_* after inhibition of cellular uptake pathways (e.g. endocytosis, macropinocytosis, and ASBT-mediated transport) confirmed that these routes played important roles in improving the oral bioavailability of ELNE#7 (Figure 3(A,B)). Another explanation for the improved bioavailability involves the avoidance of recognition by P-gp or possible suppressive effects of pharmaceutical excipients incorporated into ELNE#7 (e.g. Capryol 90, Tween 80, and Transcutol HP).

## 4. Conclusions

This study showed that the DCK–PA-anchored nanoemulsion vehicle incorporating ETP/LMC (1:5, w/w) significantly enhanced the intestinal permeability and oral bioavailability of ETP. The *P_app_* value of ELNE#7 was 5.16- and 2.25-fold greater than those of ETP dispersed in water and ETP emulsion, respectively. Moreover, the oral bioavailability of ELNE#7 was 1752% and 224% higher than those of ETP dispersed in water and commercial ETP emulsion, respectively. In addition, the results of the intestinal transport mechanistic study suggested that mechanisms such as clathrin/caveola-mediated endocytosis, macropinocytosis, and ASBT-mediated pathways contributed to the enhanced permeability and oral bioavailability. Moreover, P-gp-mediated efflux did not appear to significantly impede the permeation of ELNE#7. In addition, the cytotoxicity study demonstrated enhanced toxic effects of ELNE#7 on LLC and A549 cells in comparison with ETP emulsion and ETP-IV. Taken together, these observations supported the improved permeability and bioavailability of ETP, which is expected to show better therapeutic potential compared with ETP emulsion.

## Supplementary Material

Supplemental Material
