# Supplementary material for: Enhanced oral bioavailability of an etoposide multiple nanoemulsion incorporating a deoxycholic acid derivative–lipid complex
Source: Drug Deliv. 2020 Oct 27;27(1):1501–13. doi: 10.1080/10717544.2020.1837293 (PMC7594851; doi:10.1080/10717544.2020.1837293)
Supplement: Supplemental Material [file IDRD_A_1837293_SM2931.docx]

**Supporting Information**

**Enhanced oral bioavailability of an etoposide multiple nanoemulsion incorporating a deoxycholic acid derivative–lipid complex**

Saurav Kumar Jha^a^, Hee-Soo Han^b^, Laxman Subedi^a^, Rudra Pangeni^c^, Jee Young Chung^d^, Seho Kweon^e^, Jeong Uk Choi^f^, Youngro Byun^e^, Yong-Hee Kim^b,d^, Jin Woo Park^a,c^

^a^ Department of Biomedicine, Health & Life Convergence Sciences, BK21 Four, Mokpo National University, Jeonnam 58554, Republic of Korea

^b^ Department of Bioengineering, Institute for Bioengineering and Biopharmaceutical Research, BK 21 Plus Future Biopharmaceutical Human Resources Training and Research Team, Hanyang University, Seongdong-gu, Seoul 04763, Republic of Korea

^c^ College of Pharmacy and Natural Medicine Research Institute, Mokpo National University, Muan-gun, Jeonnam 58554, Republic of Korea

^d^ Department of Bioengineering, Hanyang University, Seongdong-gu, Seoul 04763, Republic of Korea

^e^ Department of Molecular Medicine and Biopharmaceutical Science, Graduate School of Convergence Science and Technology, College of Pharmacy, Seoul National University, Seoul 08826, Republic of Korea

^f^ College of Pharmacy, Chonnam National University, Gwangju 61186, Republic of Korea

**Supplemental data**

**Table S1.** Compositions of selected w/o/w nanoemulsions with incorporation of ETP or ETP/LMC.

| Formulation code | ETP (mg) | ETP/LMC (mg) | Water (mg) | Capryol 90 (mg) | Labrasol:Transcutol HP (1:2, w/w) (mg) | Tween 80 (mg) | DCK-PA (mg) | TPGS (mg) | Water (mg) |
| --- | --- | --- | --- | --- | --- | --- | --- | --- | --- |
| ETP dispersed in water | 20 |  |  |  |  |  |  |  | 1000 |
| ETP/LMC solution |  | 120 |  |  |  |  |  |  | 1000 |
| ETP in 0.3% NaCMC | 20 |  |  |  |  |  |  |  | 1000 |
| ETP/LMC in 0.3% NaCMC |  | 120 |  |  |  |  |  |  | 1000 |
| ENE | 20 |  |  | 41.6 | 500 | 100 |  |  | 200 |
| ELNE#1 |  | 120 | 190 | 41.6 | 500 | 100 |  |  | 200 |
| ELNE#2 |  | 120 | 190 | 41.6 | 500 | 100 |  | 100 | 200 |
| ELNE#3 |  | 120 | 190 | 41.6 | 500 | 100 |  | 200 | 200 |
| ELNE#4 |  | 120 | 190 | 41.6 | 500 | 100 |  | 400 | 200 |
| ELNE#5 |  | 120 | 190 | 41.6 | 500 | 100 | 36 |  | 200 |
| ELNE#6 |  | 120 | 190 | 41.6 | 500 | 100 | 72 |  | 200 |
| ELNE#7 |  | 120 | 190 | 41.6 | 500 | 100 | 144 |  | 200 |

Abbreviations: w/o/w, water-in-oil-in-water; ETP, etoposide; LMC, low-molecular-weight methylcellulose; ETP/LMC, ETP and LMC inclusion complex; ELNE, ETP/LMC incorporated nanoemulsion; NaCMC, sodium carboxymethyl cellulose; TPGS, d-alpha-tocopherol polyethylene glycol succinate; DCK, *N*^α^-deoxycholyl-l-lysyl-methylester; PA, 1,2-didecanoyl-sn-glycero-3-phosphate (sodium salt); DCK-PA, an ionic complex of DCK and PA.

**Table S2.** Inhibitors and concentrations used in the transport study, listed with their functions.

| Inhibitor | Concentration | Function |
| --- | --- | --- |
| Actinomycin D | 3.2 μM | Inhibitor of ASBT-mediated transport |
| Clofazimine | 10 μM | Inhibitor of OSTα/β blocking the transport of bile acid across the basolateral membrane |
| Chlorpromazine | 32 μM | Inhibitor of clathrin-mediated endocytosis |
| MBCD | 10 mM | Inhibitor of caveola/lipid raft-mediated endocytosis (cholesterol depletion) |
| Genistein | 0.1 mM | Inhibitor of caveola/lipid raft-mediated endocytosis (broad inhibitor of protein tyrosine kinase) |
| Amiloride | 0.1 mM | Inhibitor of macropinocytosis |
| Brefeldin A | 90 μM | Inhibitor of ER/Golgi pathway |
| Cys A | 10 µM | Inhibitor of P-gp-mediated efflux |

Abbreviations: ASBT, apical sodium-dependent bile acid transporter; OSTα/β, organic solute transporter α and β; MBCD, methyl-β-cyclodextrin; ER, endoplasmic reticulum; Cys A, cyclosporine A; P-gp, P-glycoprotein.

**Table S3.** Aqueous solubilities of a physical complex of ETP and LMC and effective permeabilities of ETP and ETP/LMC or ETP and ETP/LMC-loaded nanoemulsion.

| Test material | Aqueous solubility (μg/mL) | Effective permeability (*P_e_*, × 10^-6^ cm/s)^a^ |
| --- | --- | --- |
| ETP | 230 ± 29.4 | 0.251 ± 0.063 |
| ETP/LMC (1:1) | 3,039 ± 219 | 1.44 ± 0.750 |
| ETP/LMC (1:5) | 9,533 ± 28.8 | 2.34 ± 0.676 |
| ETP/LMC (1:10) | 1,641 ± 3.05 | 1.16 ± 0.120 |
| ETP/LMC (1:1)-NE | - | 4.71 ± 0.727 |
| ETP/LMC (1:5)-NE | - | 8.77 ± 0.722 |
| ETP/LMC (1:10)-NE | - | 5.52 ± 0.593 |

^a^Effective permeability (*P_e_*) through an artificial intestinal membrane after dissolution of ETP or ETP/LMCs in water, or incorporation into the nanoemulsion.

Abbreviations: ETP, etoposide; LMC, low-molecular-weight methylcellulose; ETP/LMC, ETP and LMC inclusion complex; ETP/LMC-NE, ETP/LMC-loaded nanoemulsion.

Each value represents the mean ± standard deviation (*n* = 6).

**Table S4.** Particle sizes, polydispersity indices, and zeta potentials of PA, DCK, DCK-PA, and ELNE formulations.

| Test material | Particle size (nm) | Polydispersity index | Zeta potential (mV) |
| --- | --- | --- | --- |
| PA | 276 ± 3.67 | 0.330 ± 0.008 | –83.4 ± 3.87 |
| DCK | 263 ± 42.4 | 0.717 ± 0.107 | 29.4 ± 0.576 |
| DCK-PA | 570 ± 19.6 | 0.195 ± 0.043 | –28.0 ± 0.212 |
| ETP dispersed in water | 1,531 ± 51.6 | 0.949 ± 0.045 | - |
| ETP/LMC solution | 384 ± 3.52 | 0.387 ± 0.061 | –6.54 ± 0.715 |
| ELNE#1 | 189 ± 10.3 | 0.377 ± 0.015 | –14.6 ± 1.68 |
| PA-ELNE#7 | 356 ± 37.0 | 0.421 ± 0.122 | –47.4 ± 1.95 |
| ELNE#7 | 172 ± 5.70 | 0.290 ± 0.005 | –11.1 ± 0.915 |

Abbreviations: PA, 1,2-didecanoyl-sn-glycero-3-phosphate (sodium salt); DCK, *N*^α^-deoxycholyl-l-lysyl-methylester; DCK-PA, an ionic complex of DCK and PA; ETP, etoposide; LMC, low-molecular-weight methylcellulose; ETP/LMC, ETP and LMC inclusion complex; ELNE#1, ETP/LMC-loaded nanoemulsion; PA-ELNE#7, PA-incorporated ELNE; ELNE#7, DCK-PA complex-incorporated ELNE.

Each value represents the mean ± standard deviation (*n* = 6).

**Table S5.** Pharmacokinetic parameters of ETP in rats after oral administration of ES, ELS, ETP-E, or ELNE#7.

| Test material | ETP-IV | ETP dispersed in water | ETP in 5% DMSO | ETP/LMC solution | ETP emulsion | ELNE#7 |
| --- | --- | --- | --- | --- | --- | --- |
| Administration | IV | Oral | Oral | Oral | Oral | Oral |
| Dose of ETP (mg/kg) | 20 | 20 | 20 | 20 | 20 | 20 |
| T_max_ (h) | - | 0.88 ± 0.25 | 1.75 ± 0.289 | 0.50 ± 0.00 | 2.25 ± 1.26^*,$$^ | 1.00 ± 0.00 |
| C_max_ (ng/mL) | 11,676 ± 750 | 41.8 ± 6.36 | 67.6 ± 19.3 | 110 ± 17.7^**,#^ | 173 ± 17.1^***,###,$$^ | 582 ± 28.1^***,###,$$$,&&&^ |
| AUC_last_ (ng·h/mL) | 5,173 ± 484 | 89.7 ± 45.5 | 315 ± 59.7 | 337 ± 52.7^*^ | 705 ± 153^***,##,$$^ | 1,579 ± 159^***,###,$$$,&&&^ |
| AUC_inf_ (ng·h/mL) | 5,480 ± 753 | 307 ± 165 | 486 ± 59.3 | 483 ± 140 | 1,078 ± 498^**,#,$^ | 1,646 ± 201^***,###,$$$^ |
| Bioavailability (%) | 100 | 1.74 ± 0.880 | 3.04 ± 0.577 | 6.51 ± 1.02^*^ | 13.6 ± 2.96^***,###,$$^ | 30.5 ± 3.08^***,###,$$$,&&&^ |

Abbreviations: ETP, etoposide; DMSO, dimethyl sulfoxide; LMC, low-molecular-weight methylcellulose; ETP/LMC, ETP and LMC inclusion complex; ETP emulsion, ETP incorporated in emulsion containing PEG, glycerol, and citric acid anhydrous; ELNE#7, ETP/LMC-loaded and DCK-PA incorporated nanoemulsion; IV, intravenous; T_max_, time to reach maximum plasma concentration; T_1/2_, half-life of plasma concentration; C_max_, maximum plasma concentration; AUC_last_, area under the plasma concentration–time curve from zero to the time of the last measurable plasma concentration; AUC_inf_, area under the plasma concentration–time curve from zero to infinity. Each value represents the mean ± standard deviation (*n* = 4). Bioavailability (%) = (AUC_last_, _oral_/Dose_ETP, oral_)/(AUC_last_, _IV_/Dose_ETP, IV_) × 100. **p* < .05, ***p* < .01, ****p* < .001 compared to ETP dispersed in water. ^#^*p* < .05, ^##^*p* < .01, ^###^*p* < .001 compared to ETP in 5% DMSO. ^$^*p* < .05, ^$$^*p* < .01, ^$$$^*p* < .001 compared to ETP/LMC in water. ^&&&^*p* < .001 compared to ETP emulsion.


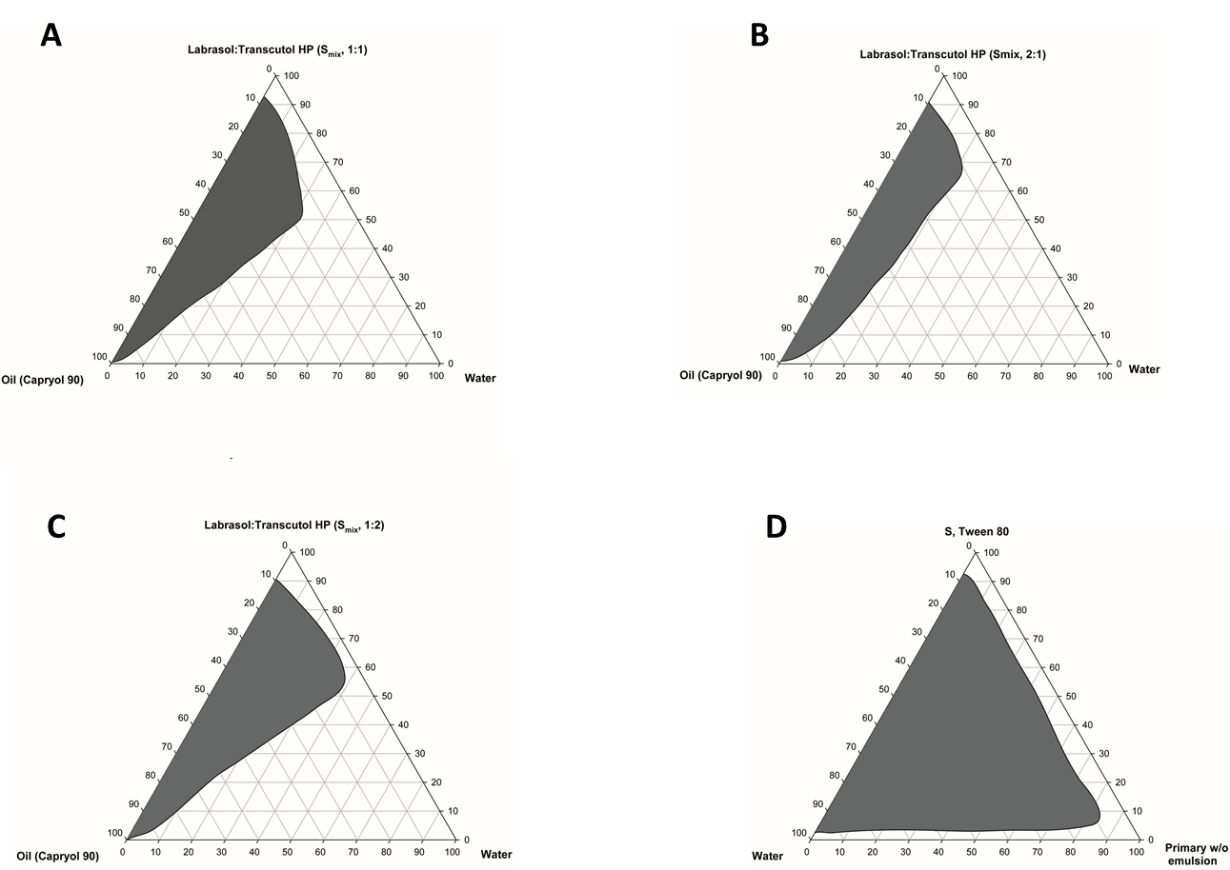


**Figure S1.** Pseudo-ternary phase diagrams of water-in-oil (w/o) nanoemulsion region of Capryol 90 (oil phase), water (aqueous phase), and a mixture of Labrasol (surfactant) and Transcutol HP (co-surfactant) with different ratios of surfactant and co-surfactant (S_mix_): (A) S_mix_ 1:1; (B) S_mix_ 2:1; (C) S_mix_ 1:2; and (D) pseudo-ternary phase diagram of water-in-oil-in-water (w/o/w) nanoemulsion.
